# Radical resection of a pelvic angiomyxoma guided by 3D reconstruction: a two-stage approach – a case report

**DOI:** 10.1097/RC9.0000000000000950

**Published:** 2026-09-24

**Authors:** Julio E. Hernández Ug, Sylwia Bilas Sudol, Celia Aguilar Martínez, José J. Pozo Kreilinger, José T. Borjas Gómez, María I. Prieto Nieto

**Affiliations:** a Department of General and Digestive Surgery, Hospital Universitario La Paz, Madrid, Spain; b Department of Pathology, Hospital Universitario La Paz, Madrid, Spain; c 3D Laboratory Unit, Hospital Universitario La Paz, Madrid, Spain; d Peritoneal Oncology and Sarcoma Surgery Unit, Hospital Universitario La Paz, Universidad Autónoma de Madrid, Madrid, Spain

**Keywords:** 3D reconstruction, aggressive angiomyxoma, case report, partial sacrectomy, pelvic tumor, surgical planning

## Abstract

**Introduction:**

Aggressive angiomyxoma is a rare, locally infiltrative mesenchymal tumor that predominantly affects women and occurs only exceptionally in men. Surgical management of large pelvic lesions is technically challenging because of the complex anatomy and close relationship to critical adjacent structures. Three-dimensional reconstruction may improve preoperative planning and intraoperative orientation in such cases.

**Case presentation:**

A 58-year-old man presented with a large pelvic mass diagnosed as an aggressive angiomyxoma. The preoperative assessment included computed tomography (CT) and magnetic resonance imaging (MRI), followed by patient-specific three-dimensional reconstruction based on CT-MRI fusion. This approach enabled detailed anatomical visualization and precise planning of the surgical strategy, including the determination of the level of sacrectomy.

**Results:**

Radical en bloc resection was performed using a two-stage approach, combining a prone position for partial sacrectomy and a supine position for abdominoperineal resection. The three-dimensional model facilitated the identification of tumor boundaries, spatial relationships, and critical vascular structures, allowing anticipation of the most technically demanding steps. Complete resection with negative margins was achieved. The postoperative course was uneventful, and the patient was discharged on postoperative day 10. No recurrence has been observed to date.

**Conclusion:**

Aggressive angiomyxoma of the pelvis in male patients requires an individualized, multidisciplinary approach. Three-dimensional reconstruction is a valuable tool for surgical planning in complex pelvic tumors, improving anatomical understanding and facilitating safe resection with negative margins.

## Introduction

Aggressive angiomyxoma (AAM) is a rare mesenchymal tumor characterized by slow growth and locally infiltrative behavior, most frequently located in the pelvis and perineum^[^1,2^]^. It predominantly affects women of reproductive age, with a female-to-male ratio of approximately 6:1^[^1^]^. Presentation in male patients may pose a significant diagnostic challenge due to the lesion’s deep location and the nonspecific nature of the initial symptoms^[^3,4^]^.

HIGHLIGHTS

- Aggressive angiomyxoma is a locally infiltrative mesenchymal tumor that predominantly affects women and is extremely rare in men.
- It usually presents as a slow-growing pelvic mass with symptoms caused by compression of adjacent structures.
- Differential diagnosis with other pelvic mesenchymal tumors is challenging, and definitive diagnosis requires histopathological examination.
- Complete surgical resection with negative margins remains the main treatment whenever feasible.
- In complex pelvic cases, three-dimensional reconstruction may play an important role in preoperative surgical planning.

AAM may resemble other pelvic mesenchymal neoplasms, making the differential diagnosis difficult. Magnetic resonance imaging (MRI) is essential for lesion characterization and assessment of the local extent. However, definitive diagnosis requires histopathological examination^[^3,5,6^]^. In cases of marked anatomical complexity, three-dimensional reconstruction may be useful in preoperative planning.

We describe the case of a giant pelvic AAM in a male patient with an unusual presentation and complex surgical management, emphasizing the role of three-dimensional reconstruction in the surgical strategy.

Our case has been reported in line with the SCARE criteria.

## Case report

We report the case of a 58-year-old man who was referred to the Urology Department for erectile dysfunction. Physical and digital rectal examinations revealed lateral displacement of the prostate by a firm, elastic mass in the prostatic region. On transrectal vesicoprostatic ultrasound, the lesion did not show an ultrasonographic pattern compatible with normal prostatic parenchyma, and a biopsy was therefore performed for histological characterization. Histopathological examination showed findings consistent with AAM.

Computed tomography (CT) and MRI demonstrated a 13 × 8 × 9.5 cm well-defined solid lesion located in the left perirectal space, adjacent to the sacral promontory and extending cranially to S1, with abundant myxoid content, marked intralesional hypervascularity, and a significant mass effect, without clear signs of infiltration of adjacent structures (Fig. 1).

**Figure 1.**
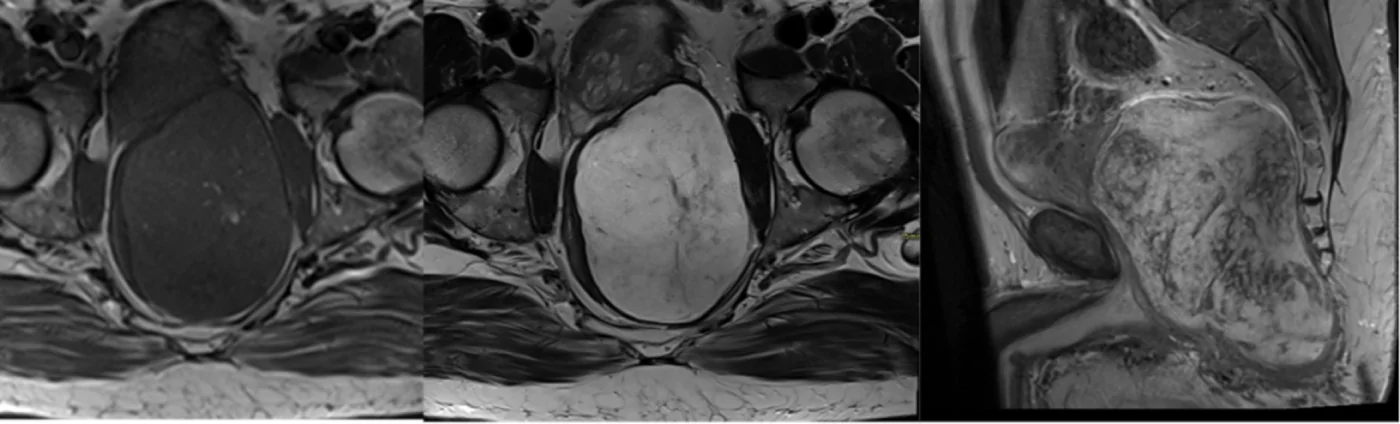
MRI showing a large, well-defined presacral mass that is hypointense on T1-weighted images and hyperintense on T2-weighted images, with intense enhancement after paramagnetic contrast. Serpentine intralesional structures suggest prominent vascular components.

After discussion by the multidisciplinary oncology board, radical surgery with curative intent was indicated.

The procedure was planned using three-dimensional reconstruction, which enabled definition of the surgical strategy and anticipation of the most technically demanding steps of the operation. The patient-specific three-dimensional reconstruction was generated using Mimics segmentation software (Materialize), combining CT-based segmentation with MRI registration, mainly sagittal T1- and T2-weighted sequences (Fig. 2). Contrast-enhanced CT allowed adequate visualization of vascular structures, while MRI helped delineate tumor boundaries, margins, and anatomical relationships.

**Figure 2.**
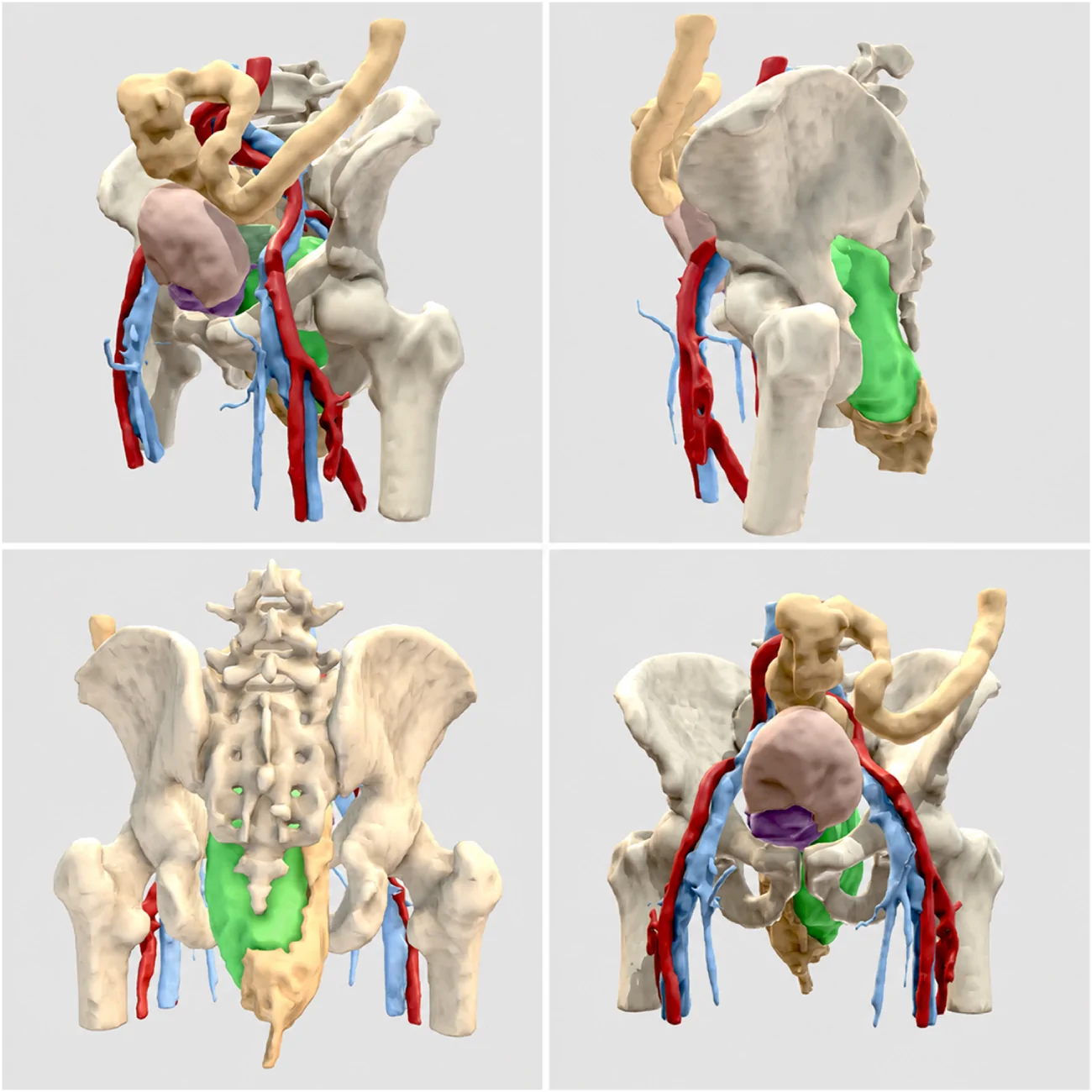
Patient-specific three-dimensional reconstruction generated from CT and MRI data. The model shows the tumor in contact with the inner surface of the sacrum, with lateral displacement of the rectum.

During the first surgical stage, with the patient in the jackknife position, partial sacrectomy up to S3 was performed using the BoneScalpel ultrasonic bone-cutting device (Misonix; Dismeval), which enabled precise and effective bone resection. The patient was then placed in the supine position, and abdominoperineal resection was carried out, including en bloc removal of the rectum, sigmoid colon, and tumor mass, which was extracted through the perianal route (Fig. 3A). The procedure was technically demanding because of marked hypervascularity and adhesions to the sacrum. A terminal colostomy was created in the left iliac fossa, and pelvic drains were left in place. Complete resection with negative margins was achieved. The postoperative course was uneventful, and the patient was discharged on postoperative day 10.

**Figure 3.**
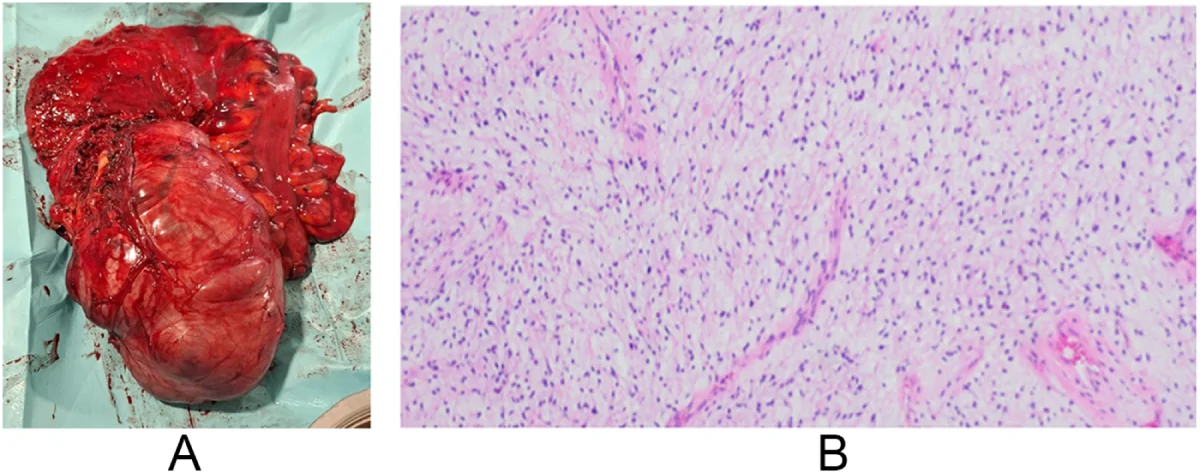
(A) En bloc resection showing a large tumor with a smooth surface and pseudoencapsulated appearance, resected in continuity with the rectum and the distal sigmoid colon. (B) Hematoxylin and eosin stain showing features of aggressive angiomyxoma, with scattered spindle and stellate cells in an abundant myxoid stroma and vessels of variable caliber, without significant cytologic atypia.

During outpatient follow-up, the patient showed progressive functional recovery. A radiological surveillance protocol was established for early detection of recurrence. At 9 months of follow-up, the patient remained free of local recurrence and distant metastases.

## Discussion

AAM is a rare mesenchymal tumor usually located in the pelvis and perineum, with locally infiltrative behavior and a high tendency toward local recurrence. It was first described by Steeper and Rosai in 1983, who introduced the term “aggressive” to emphasize its marked infiltrative capacity despite its histologically benign appearance^[^1,2,4,7^]^. The histogenesis of AAM remains uncertain, and different cellular origins have been proposed without definitive conclusions^[^1,3^]^.

Clinically, AAM usually presents as a large, slowly growing pelvic mass, often producing symptoms related to compression of adjacent structures, including urinary, genital, or gastrointestinal symptoms^[^1,2^]^.

On imaging studies, AAM usually appears as a well-defined mass that displaces adjacent structures without overt invasion^[^2,5^]^. On CT, AAM typically shows lower attenuation than muscle, reflecting its high water content and myxoid matrix, with marked heterogeneous enhancement after intravenous contrast administration^[^2^]^. MRI is the modality of choice. The tumor is typically isointense to muscle on T1-weighted sequences and hyperintense on T2-weighted sequences, with avid enhancement and a characteristic internal whorled or layered pattern after gadolinium administration. Although this pattern is not pathognomonic, it is considered highly suggestive and has been reported in approximately 80–85% of cases^[^2,5^]^.

Taken together, these radiological features and the clinical context are crucial in the differential diagnosis of other pelvic mesenchymal neoplasms, such as angiomyofibroblastoma, liposarcoma, solitary fibrous tumor, fibroepithelial stromal polyps, myxoid lipomatous tumors, and myxoid leiomyomas^[^8,9^]^.

The definitive diagnosis of AAM is established by pathological examination. Microscopically, the tumor is characterized by a loose myxoid stroma rich in mucopolysaccharides, numerous blood vessels of variable caliber, and spindle-shaped or stellate cells with scant eosinophilic cytoplasm and oval nuclei^[^3–5^]^. Nuclear atypia is typically absent, and mitotic activity is usually low, reflecting its slow-growing biological behavior (Fig. 3B). Bundles of smooth muscle adjacent to blood vessels are often seen, a characteristic histological feature of AAM^[^1,3^]^.

Immunohistochemically, tumor cells show variable expression of mesenchymal markers and are typically positive for vimentin, desmin, smooth muscle actin, and CD34, although none of these markers is specific. Estrogen receptor (ER) and progesterone receptor (PR) positivity has also been described^[^4,5^]^. These tumors typically show a low proliferative index (Ki-67), generally below 5%.

With recent technological advances, three-dimensional reconstruction has progressively been incorporated as a supportive tool in the preoperative planning of large lesions with high anatomical complexity^[^10,11^]^. Patient-specific models provide a comprehensive view of the anatomy and help clarify the spatial relationships between the lesion and adjacent structures. In complex pelvic oncologic surgery, particularly in sarcomas and other difficult-to-approach tumors, this technology may improve preoperative anatomical understanding, contribute to more precise resection planning, and facilitate anticipation of the most technically demanding steps^[^11–13^]^. However, specific evidence in pelvic soft-tissue mesenchymal tumors remains limited. In our case, three-dimensional reconstruction was particularly useful for defining the level of sacrectomy and visualizing the spatial relationships between the tumor and adjacent pelvic structures. Nevertheless, it could not reliably determine the need for multivisceral resection or whether sphincter preservation was possible. These decisions ultimately depended on direct intraoperative assessment.

Despite its potential advantages, three-dimensional reconstruction has several limitations. Its implementation requires dedicated software, high-quality imaging studies, specific technical expertise, and additional time for image segmentation and model preparation^[^10,11^]^. Furthermore, reconstruction accuracy depends on the quality and timing of the source imaging, and the model may not reliably predict microscopic invasion or the characteristics of intraoperative tissue planes^[^11,12^]^. Cost and limited availability may also restrict its routine use, particularly in centers without specialized radiological or surgical planning teams^[^10,11^]^.

Traditionally, complete surgical resection aiming to achieve negative margins whenever possible has been considered the treatment of choice. However, radical excision may be technically demanding and associated with significant morbidity because of the frequent involvement of complex pelvic floor structures^[^1,2^]^. Some series have questioned whether greater surgical radicality necessarily translates into lower recurrence rates, leading to a reconsideration of mutilating surgery, particularly when sphincteric or urogenital function may be compromised^[^14,15^]^.

Conservative and sphincter-preserving resections have been reported in selected patients with large AAMs, particularly when a safe plane of separation from the rectum and sphincteric complex can be identified, and the tumor can be removed en bloc without rupture^[^15–17^]^. Conversely, an abdominoperineal approach may be appropriate for low perirectal tumors when the border with the rectal wall is indistinct or complete resection cannot be ensured otherwise^[^18^]^. Therefore, the surgical strategy should be individualized according to tumor location, anatomical relationships, dissection planes, and expected morbidity.

In our case, the tumor occupied a substantial proportion of the narrow male pelvis, with no clear dissection plane between the mass and the anterior surface of the sacrum. Therefore, partial sacrectomy at the S3 level was performed to create sufficient space for tumor mobilization and facilitate complete resection. Given the tumor’s low location, marked adhesions, and the technical difficulty of dissection within the restricted pelvic space, a sphincter-preserving procedure was not considered technically or oncologically safe. Intraoperatively, there was no involvement of the bladder, its peritoneal covering, or Denonvilliers’ fascia; therefore, bladder resection was not required, allowing bladder preservation without compromising oncologic radicality.

Because a significant proportion of AAMs express ER and PR, hormonal treatment with gonadotropin-releasing hormone agonists has been described as a neoadjuvant or adjuvant strategy, or even as an alternative to surgery in selected cases^[^4,5^]^. By inducing a hypoestrogenic state, these agents may reduce tumor volume, facilitate less aggressive surgery, and decrease the risk of recurrence. However, current evidence remains limited, and further studies are needed to determine the potential value of hormonal therapy in reducing surgical morbidity in selected cases^[^5^]^.

Other therapeutic modalities, such as radiotherapy, vascular embolization, or chemical ablation with ethanol and acetic acid, have been described only anecdotally, generally in the setting of unresectable or recurrent disease^[^3,14^]^.

## Limitations

This case suggests that patient-specific three-dimensional reconstruction, combined with a two-stage surgical approach, may support the multidisciplinary management of complex pelvic tumors. Because this is a single case report, these findings are descriptive and cannot establish definitive clinical guidelines. Further studies are required before broader recommendations can be made.

## Conclusion

This case highlights the value of 3D reconstruction as an adjunct to preoperative anatomical assessment and planning for partial sacrectomy in complex pelvic tumors. However, preoperative imaging, including 3D reconstruction, may not accurately predict the extent of involvement of adjacent structures. Therefore, the final extent of resection should ultimately be guided by meticulous intraoperative assessment to achieve oncologically adequate margins while avoiding unnecessary organ resection.

## Data Availability

The data that support the findings of this study are available from the corresponding author upon reasonable request.
